# Kairine and Antipyrine in the Treatment of Fever

**Published:** 1885-06-20

**Authors:** P. R. Cortelyou

**Affiliations:** Marietta, Ga.


### KAIRINE AND ANTIPYRINE IN THE TREATMENT OF FEVER.

By P. R. Cortelyou, M. D., Marietta, Ga.

The skill of the physician is often sought to allay symptoms, and thus afford relief, even though he may not be successful in working a cure of the disease.

One of the most prominent symptoms, of many diseases, is the elevation of temperature, and consequent wasting and lowering of the vital force.

Thus it has been the desire of the physician to obtain some reliable febrifuge, and the results of every new remedy are watched with interest.

I desire, therefore, to give the result of my experience in the use of these two new remedies in controlling the fever in chronic pulmonary diseases, as phthisis pulmonalis and catarrhal pneumonia ; and I shall thus give the record of a few cases treated by kairine, and a few by antipyrine.

Kairine is a methyl-hydrate of oxyquinoline, being like quinine, a derivitive of quinoline, and given in doses of five grains every hour and a half to three hours; will lower the temperature from one-half to two degrees after the first dose.

CASE I.—W. S. came under my care September, 1884, giving the following history: Early in spring caught heavy cold, causing cough, fever, and loss of flesh. An examination found consolidation at apex of left lung, with localized rales, shortness of breath on exertion, red line about the gums, hoarseness of the voice with congestion of the vocal cords. Temperature under the tongue, 101. Several of his family died of tuberculosis. Used large doses of quinine to reduce temperature, but without effect except to disorder the stomach. Tried Fowler's solution of arsenic, but fever still continued.

September 26th. Temperature under tongue, 102. Gave kairine in 5-grain doses every three hours.

September 27th. Temperature, 100½. Patient said that, after taking four doses, head became dizzy—so reduced to 5 grains four times a day.

September 29th. Temperature, 99½. Bears medicine well, and from that time temperature reduced to 98½, and remained so for several days. Kairine was then discontinued, and fever returned. Renewed medicine, with the effect of bringing temperature down to normal.

May 18th. The disease seems to be arrested, patient being free from all fever, and has been for several months, and has gained some fifteen pounds in flesh.

CASE II.—Mrs. R——. Saw patient early in May. She gave the following history: Has had cough for some time, with pains in chest, and has been losing flesh. Examination revealed some signs of consolidation at apex of left lung. Did not see patient again until October 31, 1884. Had failed a good deal in flesh and strength. Had cough; fever every day; lung tissue breaking down. Temperature, 101½. Ordered kairine in capsules, 5 grains four times a day.

November 11th. Patient reports fever controlled while taking medicine, but that it made her head feel bad and dizzy. Fever returned when medicine was stopped. Ordered 5 grains three times a day.

November 20th. Reports fever but only day since last report.

CASE III.—G. N., aged 26 years. Saw patient January 16,

1885. Gave history of trouble dating back over one year. Cough, loss of flesh, fever, night sweats. Examination revealed evidences of consolidation at left apex, with some moist rales. Temperature under tongue, 99½. Prescribed kairine, 5 grains in capsules, four times a day.

January 19th. Medicine has controlled fever and checked night sweats. Since that time fever has been controlled, but not the sweating, although it does not occur as frequently as before taking the medicine.

CASE IV.—Mrs. S. Called to see patient January 29, 1885. Gave history of cough, and failure in strength and flesh, since last spring. Examination revealed marked disease of right lung anteriorly, with general catarrhal inflammation of smaller tubes of whole lung. Temperature under the tongue, 102½. Ordered kairine, grains 5, in capsules, three times a day.

January 30th. Temperature, 99½. January 31st, 100. February 2d, 98½. Patient then discontinued medicine on her own responsibility, because she said it made her head feel badly. The fever returned, and for a month ranged from 102 to 104, when again returned to kairine and reduced temperture from 104 to 101. At this time patient passed from under my care.

I have thus given the results from use of Kairine in a few cases, and will now give a few showing effect of Antipyrine—

"This is an artificial product, a synthetical alkaloid, obtained from coal tar, being chemically dimethytox quinine, and occuring in the form of a chloride. It is a grayish white powder with a slight tarry odor, and somewhat bitter taste ; soluble in one part to three of cold water, but requires only half of its weight of warm water." May be given in doses from eight to fifteen grains. Its peculiar action is in reducing temperature in fever.

CASE I. Mrs. ——. Phthisis pulmonalis with softening daily temperature 102 to 103. Gave antipyrine, 5 grains every hour until 30 grains had been taken ; temperature reduced to 99, and this dose, continued daily, kept the temperature there with no unpleasant effects. Some days only took 15 grains.

CASE II. Mrs. V. Pulmonary phthisis ; evidences of softening at right apex ; temperature 101½. Gave 7½ grains in solution every hour until 35 grains had been taken ; temperature reduced to 98½ with profuse sweating. From 15 to 20 grains in divided doses control temperature without any unpleasant symptoms.

CASE III. Miss C. Phthisis pulmonalis, with evidences of softening ; temperature 103. Gave antipyrine, 8 grains in solution every hour till 32 grains had been taken ; temperature reduced to 98½.

Three doses a day, of 8 grains each, control fever, but have produced some sickness at stomach.

From my experience with these remedies thus far, I have found them to be more effective than quinine in reducing fever, and not producing as unpleasant symptoms. Of the two, antipyrine has, in my hands, proved more certain and quicker in its action than kairine, and will be, I think, a valuable medicine in controlling fever in many acute diseases as in the chronic cases.

